# Climate changes and solar cycles recorded at the Holocene Paraná Delta, and their impact on human population

**DOI:** 10.1038/srep12851

**Published:** 2015-08-06

**Authors:** Juan Pablo Milana, Daniela Kröhling

**Affiliations:** 1 CONICET - Universidad Nacional de San Juan, InGeo, (5401) San Juan, Argentina; 2 CONICET - Universidad Nacional del Litoral, CC 217 (3000) Santa Fe, Argentina

## Abstract

The Paraná delta, growing at a rate of c. 2 km^2^ yr^−1^ since 6,000 yrs, is one of the most complete records of the Late Holocene in southern South America. The evolution of this 17,400 km^2^ delta enclosed in Plata estuary, can be tracked by a series of 343 successive coastal-ridges showing a c.11 years period, in coincidence with sunspot cycle, also found in some North Hemisphere coastal-ridge successions. The Paraná delta shifted from fluvial, to wave-dominated, and back to the present fluvial-dominated delta, in response to climate changes associated with wind activity correlating with South American glacial cycles. The wave-dominated windy period coincides with the activation of the Pampean Sand Sea, suggesting desert conditions prevailed on the Pampas between 5,300 and 1,700 yrs, in coincidence with scarce or absent pre-historic aborigine remains (“archeological silence”). Further warmer and less windy conditions allowed human repopulation. Results suggest that aside the solar forcing, both short and medium term climate changes controlled delta evolution. An important learning is that a slight cooling would turn the highly productive pampas, into that unproductive desert and, given the lack of artificial irrigation systems, changing present-day warmhouse into a cooling cycle might be economically catastrophic for the region.

Deltas are excellent environmental archives, as they are the main sink of river sediments. The Paraná-Plata drainage basin (Fig. 1) is the second largest in South America and the third worldwide, while average discharge rates sixth in the world. About 160 million tons of sediment are delivered annually by the Paraná to the Plata estuary, creating a subaerial delta plain that only in the previous 106 years grew 196 km^2^ [Ref. 1] creating land at a rate of 2.49 km^2^/yr [Ref. 2]. This delta is especially suitable as an archive of the evolution of a large area as it is protected from marine erosion by the Plata estuary which also restricts the delta-lobe shifting process. In spite of this protection, large environmental changes of continental scale^3^ reshaped the entire delta, as we will demonstrate. The importance of this geoarchive is crucial to understand prehistorical populations, but as it is located within the most industrialized and developed region of South America, is fundamental to build environmental models of the region to forecast possible crisis related to climate changes.

## The Paraná Delta

**Figure 1:**
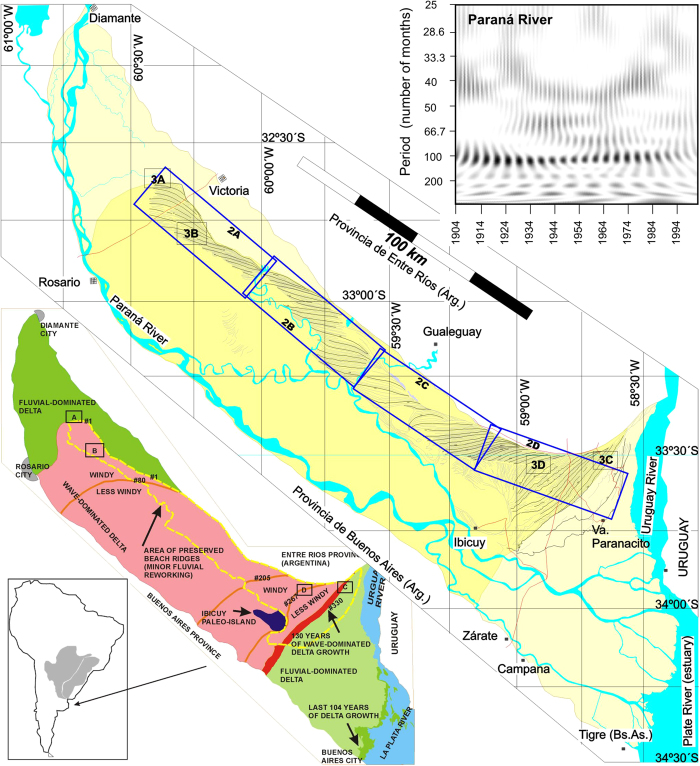
General Map of the Paraná Delta showing major delta growth stages and the continuous coastal ridges succession preserved. Boxes labeled 2A–D ar shown in detail in Fig. 2; small boxes labeled 3A-D give location of images of Fig. 3. The upper right corner box shows the real part of the continuous Morlet wavelet spectra of the Paraná river (courtesy of A. Pasquini^21^), with dark grays corresponding to high values of the transform coefficients (power), showing a strong decadal periodicity. *Map elaborated by the authors using CONAE imagery and graphical software CorelDraw 12.0 TM*.

This delta is being built within the La Plata estuary, and while marine energy does not affect it significantly, the surface is subjected to a permanent fluvial reworking exerted by the Paraná River. Main fluvial channels tend to be located along the southwest margin (Fig. 1) and reworking is almost absent along the north-east margin. Present day delta system grows at a sustained rate of 249 ha/yr [Ref. 2], and does not show any sign of longshore drift. Major flooding today is not fluvial but related to the effect of the “Sudestada” storms which could raise water level up to 4 m, and may be more active during warmer climate stages^4^. The other important wind is the Pampero that results from normal polar fronts, and as it blows strongly from the south is expected to create a strong longshore drift to the north. The evidence of a strong influence of oblique winds (Pampero) on the delta front is suggested by the linearity of coastal ridges that composed successive delta fronts, which do not form today.

The Paraná delta, grows today from a terraforming nucleus formed when a large amount of buoyant vegetal debris strands in shoals of the estuary, usually near the exit of a distributary channel. It follows the silting of the vegetal network and the progressive island expansion until starts interacting with neighboring islands. At this stage, silting locates along the channels and so, creating islands with higher margins and low swampy centers. This process did not occur when delta front was straight, but intermediate versions of it, are portrayed by delta fronts defined by segmented ridges, showing curved segment edges, indicating the position of small distributary channels. Thus, the absence/presence and the shape of coastal ridges make possible to reconstruct the environmental evolution of this delta.

**Figure 2:**
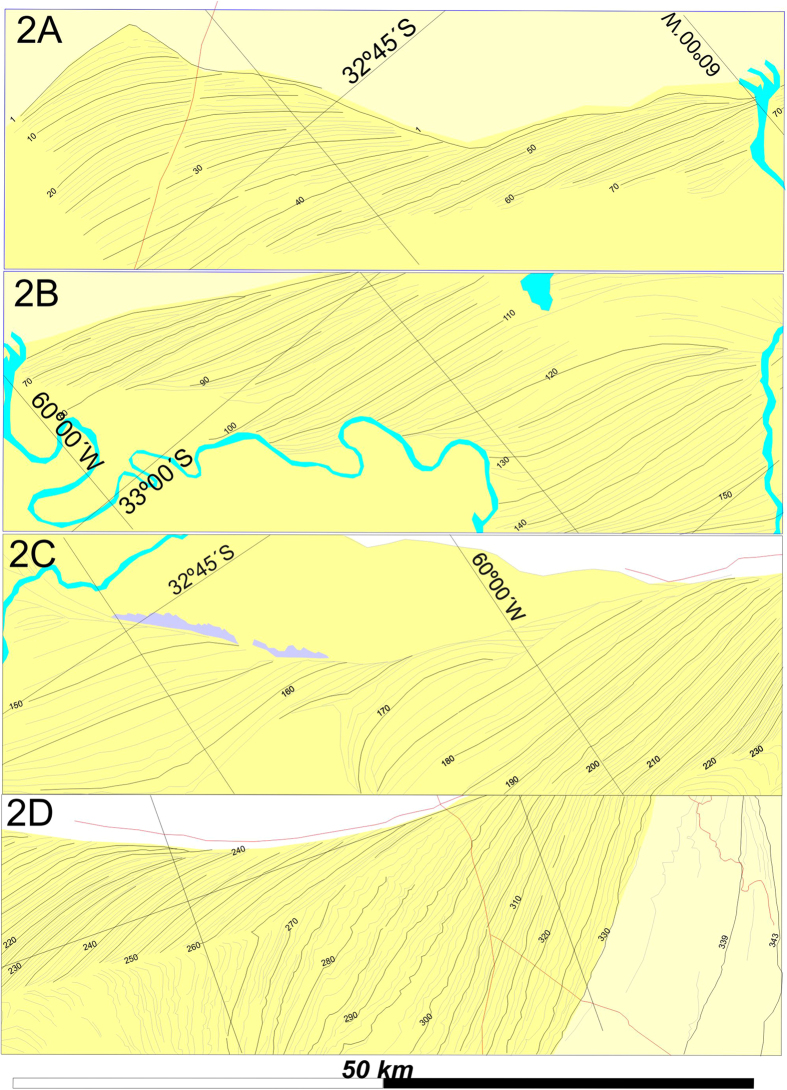
Detailed coastal ridge mapping showing the development of the succession and erosion & hiatus surfaces shown in Fig. 3. Box 2C shows the interference produced by a paleoisland, while largest paleoisland (Ibicuy^6^,^7^) is portrayed on Fig. 1, and its effect on ridge morphology is observed by sharp curves of box 2D. Box locations on Fig. 1. *Map elaborated by the authors using CONAE imagery and graphical software CorelDraw 12.0 TM*.

Our study was based on the detailed mapping based on aerial photographs and high-resolution satellite imagery, plus field control, of the remains of the original delta structure. The low degree of reworking along the northeast margin allowed mapping a continuous succession of 343 consecutive coastal ridges (Figs 1 & 2), and their significance is the main focus of this paper. The recognition of coastal ridges in the Paraná Delta is not new, and they have been described by several authors as Holocene beach deposits^5^,^6^,^7^,^8^, although there was not a detailed mapping, integration, numbering and tracing of these conspicuous ridges (Fig. 2), which are well-defined in remote imagery, but very subtle in the field.

## Age Constraining and Ridge Evolution

Most researchers agree the present delta started to evolve after the last sea-level maximum (estimated to be about 5 m higher than present sea-level) at c. 6,000 yrs BP^6^,^7^,^9^, when sea reached up to Diamante city (Fig. 1). Two major changes occurred during construction of this delta, which cannot be ascribed to sea-level. The first is the apparition of coastal ridges as a significant structural part of the delta, and the second is their almost complete disappearance. It is widely accepted this type of coastal ridges is wave-generated, thus the only explanation for these changes is variations of wind strength or direction, within the Plata estuary.

**Figure 3:**
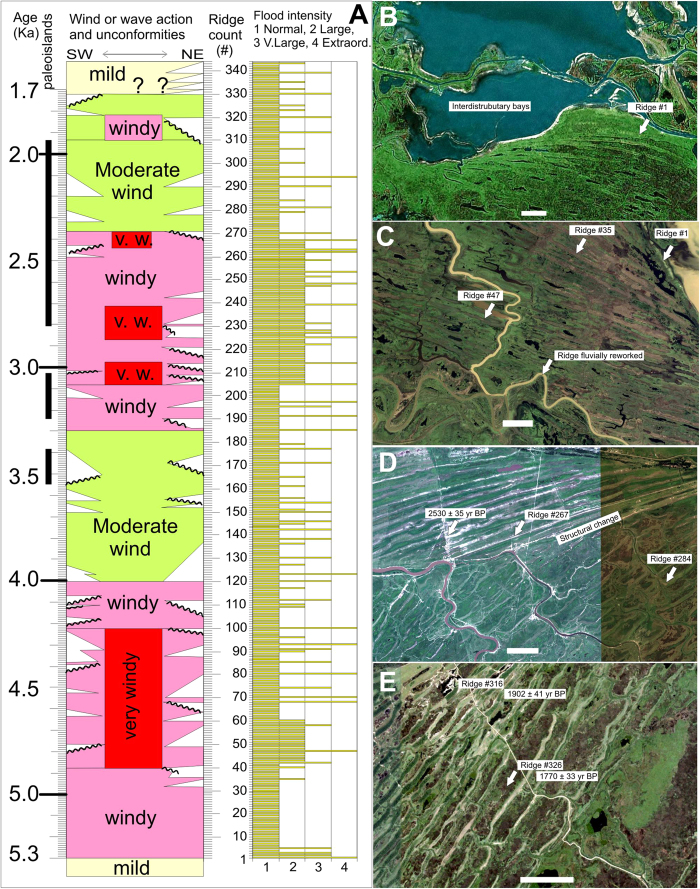
Evolution of the wave-dominated delta with captions of environmental changes. (**A**) Plot of ridge unconformities, wind intensity (linearity), river flood intensity (ridge size) and delta front interaction with paleoislands against preferred time-frame. Ridges after #330 do not follow same time-scale. (**B**) Change from the fluvial-dominated delta with inter-distributary bays to a wave-dominated delta at ridge #1, (**C**) Convergence of younger ridges with ridge #1, and fluvial reworking. (**D**) Change from linear to segmented and curved ridges, showing location of ^14^C dating suggesting climate amelioration started at c. 2,400 yrs BP. (**E**) Last ridges of the continuous 330 succession, with location of published ^14^C dates^6^,^7^, marking the onset of present-day mild conditions. Scale bar is 1 km in each scene.

The first change, a switch from a fluvial-dominated to a wave-dominated delta, evidenced by the apparition of coastal-ridges occurred soon after the delta initiated. The first ridge (#1, Fig. 2A), has provided several bivalve shells (*Erodona mactroides*) dated by different authors by ^14^C yielding ages of 6,440 ± 110; 6,030 ± 140; 5,871 ± 42; 5,690 ± 170; 5,610 ± 110; 5,280 ± 100^6^,^7^,^8^ yrs BP. These varied ages suggest the #1 ridge is stratigraphically complex, evidenced in our mapping by the convergence of ridges #2 until #80 (Figs 1 and 2A,B) towards this same ridge. Due to the age uncertainty, and the fact many complex coastal ridges contain reworked shells of unknown age we used the youngest date (c. 5,300 yrs. BP) for determining the starting time for ridge progradation, which as discussed further is consistent with other high-resolution climate records of South America. We also used the youngest date obtained on this ridge for the time of wave-dominated delta initiation, as this ridge collected reworked shells of previously deposited units, for at least 900 years, our estimated time span between ridges #1 and #80. During the wave-dominated delta, a delta front was formed and delta-foresets were recorded by Colombo *et al*. (2007)^10^ using ground penetrating radar. The second major change is a return to a fluvial-dominated delta, determined by a rather abrupt end to this long beach-ridge succession. This change is estimated at c. 1,720 yrs BP based on two ^14^C dates of 1,770 ± 33 and 1,902 ± 41^5^,^6^,^7^, from ridges #316 and #326 respectively (Fig. 2D & 3E) located close to the last ridge (#332) of the succession. Several erosive surfaces and hiatuses are recognized along this ridge succession at both extremes of some ridge packages (Figs 2 & 3), that we interpret as the effect of changes of locus of sediment delivery point (channel mouth) resulting in transient coastal erosion. We avoided these unconformities (lack of continuity) by following the problematic ridges laterally until a point the ridge truncation disappeared. Unconformities are represented in Fig. 3, along with the variation of two ridge characteristics: linearity and size.

We interpret ridge linearity as the enhanced effect of longshore drift. In moments of strong wind conditions the enhanced drift was capable to close all minor distributary channels mouths by building a continuous ridge, something that does not occur today here, but observed in many wave-dominated deltas worldwide. Moderate wind conditions are suspected when ridges are segmented and quite curved at segment ends, and milder conditions are shown by ridges transitional to the present no-ridge period.

Ridge size is related to a combination of width and height that makes some ridges outstanding, even during floods when smaller ones are submerged. We interpret “size” as related to the availability of more sediment to build the ridge, and due to the fact sediment is brought by the river, we suggest this relates to enhanced fluvial transport, and hence periods of different river flooding intensities. Size could be also the result of wave reworking, and a long-lived discussion of coastal ridge origin produced by sea-level variations exists. However, we assume Paraná ridges were formed as those described in the artificially induced Brazos river wave-dominated delta, where each coastal ridge was caused by extraordinary river floods^11^ instead of sea level or wave energy changes. We used the Brazos river delta model, as it is the one that best recorded ridge formation in an artificially induced river within the instrumental period. While wave energy redistributed the sand along ridges, the variable sediment input supplied, would depend on river flooding. The evolution of wind and river flooding based on ridge linearity and sizes are represented in Fig. 3.

After ridge succession end at c. 1,720 yrs BP, a group of poorly structured ridges was recognized well separated from the previous continuous succession, that are not included in this analysis. The higher ground created by these ridges allowed settlement of the largest town within the delta (Villa Paranacito), and it might be possible these ridges were the result of the Little Ice Age (LIA) cooling period.

## Correlating the Delta, Sand Seas and Glaciers

**Figure 4:**
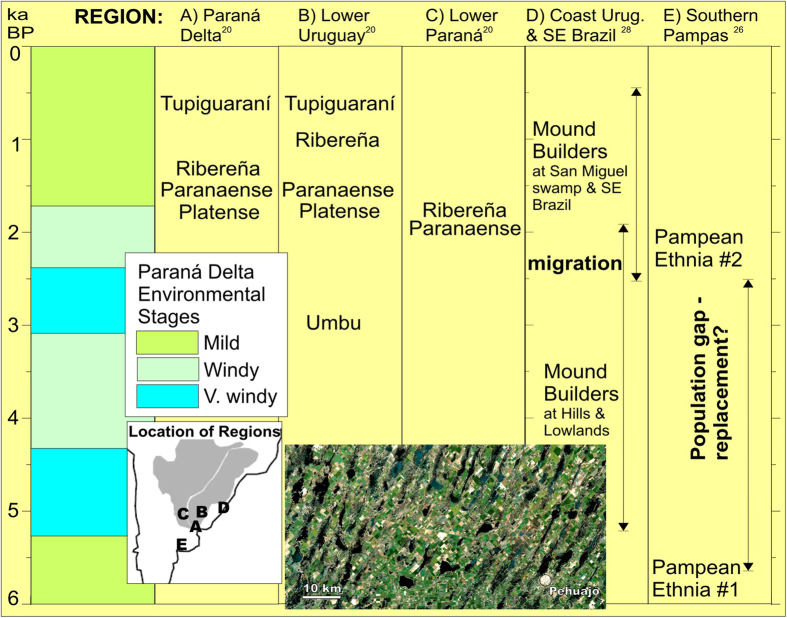
Estimated times of habitation of aborigine cultures in different regions around the Paraná Delta according to various authors^20^,^26^,^27^. Approximate location of culture regions are shown in the inset map. The scene, near Pehaujó town portrays today productive croplands over large draas (6km wavelength), reactivated during the mid-Holocene. Pehuajó is at 35°49’S, 61°54’W at the core Buenos Aires, the wealthiest state of Argentina.

Delta major structural changes indicate a period of increased wind energy lasting from c. 5,300 to 1,720 yrs BP. Due to this delta is enclosed within the Plata estuary and a northeastward sand-drift was needed to shape these ridges (Fig. 1), waves must be generated within the estuary by N to NE directed winds, perhaps like the “Pampero” windstorms. We believe that climate changes recorded here are not merely a local or regional phenomenon. There is significant evidence of a major reactivation of the Pampean Sand Sea during the mid-Holocene, and several authors extracted a regional sand wind-drift from today stabilized dunes related to N-NE directed winds, while this dry, windy period was centered around 3,500 years BP, with the last dunes active until about 2,000 years BP^12^,^13^,^14^,^15^,^16^,^17^. While there is not a complete agreement on the exact timing of this dry, sand-sea active period^18^, the few available dates show strong coincidences with our windy delta period. Anybody can see today these dormant large dunes and draas below the rich cropland of the Pampas (Fig. 4), just like the Damocles sword.

We can bracket the initiation of the windy period between 6,440 and 5,280 yrs BP, but we suggested an age of c. 5,300 yrs BP using the relation between glacial growth and wind activation^19^, as glaciated mountains generate higher barometric contrasts, creating stronger winds. Onset of windy conditions at the delta might be coincident with the growth of tropical glaciers dated on South American ice-cores to 5,300 yrs BP (see discussion of Thompson *et al.* 2006 and others on this age^3^). The timing of the switch back to the present fluvial-dominated delta can be estimated quite precisely at c. 1,720 yrs BP. This environmental change is coeval with a rapid decay of tropical mountain ice bodies, continentally and globally, at about A.D. 300, as recorded in most tropical ice cores^3^. Besides, archeological studies indicate that local human re-settlement commenced c. 2,000 yrs BP^20^: coincidence or climate amelioration? Considering these possible dates, we tested them against the delta growth rate. The three fluvial, wave and fluvial dominated phases, account for roughly 3080; 9740; and 4280 km^2^ respectively (Fig. 1), yielding growth rates of 2.8, 2.8 and 2.4 km^2^ yr^−1^ for these three major stages, defined by ages of c. 6,400; 5,300; 1,720 yrs BP and today. The decreasing growth rate through time might be due to the increasing depth of the Plata estuary. The area between the dated ridges at 1,770 and 1,930 yrs BP, suggest a growth rate of 2.1 km^2^yr^−1^, while the present rate obtained from historical coastal maps is 1.85 km^2^yr^-1^ 1 and 2.49 km^2^yr^−1^ [Ref. 2].

Assuming a constant growth and estimated dates for wave-dominated delta existence, the 330 ridges contained there imply an average time of c. 10.8 years for individual ridge formation, also consistent with the pseudo-decadal periodicity found in the Paraná river discharge^21^,^22^ (Fig. 1). This periodicity is consistent with the sunspot cycle, which is the most conspicuous cycle found in high resolution sediment archives (usually varves) of any age^23^,^24^. And, it is the period found for other high-resolution coastal ridge studies on the Mediterranean coast^25^. Having one of the largest drainage basins of the world, and a strongly seasonal hydrograph, the Paraná would be well suited to reflect periodic climate shifts. We tested our time-frame against the published ^14^C age of 2,530 ± 35 yrs BP^6^,^7^ for ridge #265. If ridge #1 is 5.3 ka and average ridge formation period is 10.8 yr, ridge #265 would yield an age of 2,597 yrs, which is fairly consistent with published dating. With these three independent controls of the proposed chronology (internal ridge dating, surface growth rate, and similarity of past and present flooding recurrence), added to a good correlations to other continental-scale environmental processes, we are quite sure about the proposed chronology.

## Importance for Human Life: Warming vs. Cooling

The evidence of environmental change in the Paraná Delta casts light on several archeological aspects of the Paraná-Plata, and the Pampean region. Our reconstruction suggests climate amelioration took place in two stages: the first reflected from the change observed at ridge #267 (2.57 ka) from very windy to moderate windy conditions, and the second when ridge succession abruptly ends at 1.72 ka. According to several workers, the Paranaense, Platense and Ribereña cultures began in the Lower Paraná, Lower Uruguay and on the Delta just after 2 ka BP^20^ (Fig. 4). At the Pampas, some researchers have proposed a population replacement based on significant differences in cranial remains of aborigines^26^, in coincidence to the delta wind-dominated interval. Additional evidence of climate forcing on human settlement between 2.5 and 1.7 ka, comes from the well known ‘*mound-builders*’ culture of East Uruguay and Southeast Brazil^27^. Between 2.5 and 2 ka, an important shift of habitation or utilization of those mounds occurred^28^. Lagoons near mound sites, like the Castillos, suggest an increase in continental water input to the lagoon at c. 2 ka, while at the Negra lagoon cores, grass phytoliths disappear abruptly near 1.7 ka, suggesting the onset of a warmer and wetter period^28^. Also, peak occupational periods on mounds marginal to these lagoons occurred between 2 and 1.6 ka.

At the lower Paraná river and surrounding plains, many authors did not find good match between human occupation periods and sea-level changes^20^,^26^,^27^, while the climate evolution revealed by the Paraná Delta provides an understandable explanation for migrations that would be difficult to understand. This is because there is an environmental match between the wave-dominated delta and sand sea reactivation; enforcing the idea the Pampas were probably a hostile environment during windy periods. Wind that probably had implications for human settlement was probably also related to restricted water availability, due to its correlation with periods of aridity^13^,^14^.

The water problem in the pampas is well portrayed by today sustained increase of precipitations, but also with historical data collected in relation to the recent LIA. Historical documents show that travelers crossing the Pampas experienced problems in finding water during the epoch locally ascribed to the LIA^13^,^14^, whereas today the Pampas are dotted with numerous endorheic lakes (Fig. 4) and small streams. We expect the LIA was an epoch milder than the windy period recorded by the wave-dominated delta, given the fact that after ridge #330, or 1,720 years BP only discontinuous and irregular coastal ridges are found and among them, we believe a small group (ridges #339 to #343) might represent the LIA at Paraná Delta (Fig. 1). Historical chronicles explain therefore why human remains were scarce in the pampas throughout the windy period recorded in the delta between 5.3 and 1.7 Ka due to its close relation to water availability, and it is not difficult to imagine a windy mid to late -Holocene when the Pampas were a barren, sandy, semi-desert plain. The climate amelioration indicated by the Paraná Delta not only made the Pampas a more favorable place for human habitation, but is the sole reason for today’s high agricultural productivity, since local crops depend on natural rainfall.

While our findings help solving the debate of whether the mid-Holocene was humid or dry^18^, they suggest an inter hemispheric connection as well. In particular, it is interesting to note that several pollen-based climate reconstruction of the western Mediterranean found arid or steppic conditions well developed from 5.3 until 1.7 ka, in coincidence with the Paraná Delta, and also the 11-year cycle is observed. The Paraná Delta data reinforces early conclusions of glacial growths between 5.3 and 2.0 ka^3^, found in ice-core datasets, suggesting wind was an important climate variable for the mid to late-Holocene. Results also suggest that present warm house favors human activity at the Pampean region, while cooling would create a catastrophic scenario due to the lack of artificial irrigation and of possible groundwater sources. This conclusion is opposite to what IPCC laureate report^29^ predicts for southern South America, indicating the need of conciliating theoretical models with true geo-archives.

## Additional Information

**How to cite this article**: Milana, J. P. and Kröhling, D. Climate changes and solar cycles recorded at the Holocene Paraná Delta, and their impact on human population. *Sci. Rep.* **5**, 12851; doi: 10.1038/srep12851 (2015).
